# Modulation of dendritic cell function by the radiation-mediated secretory protein *γ*-synuclein

**DOI:** 10.1038/cddiscovery.2015.11

**Published:** 2015-07-27

**Authors:** S-M Kang, M-H Kim, K-H Song, S-Y Jung, J Ahn, S-G Hwang, J-H Lee, D-S Lim, J-Y Song

**Affiliations:** 1 Division of Radiation Cancer Sciences, Korea Institute of Radiological and Medical Sciences, Seoul 139-706, Republic of Korea; 2 Department of Biotechnology, CHA University, Seongnam 463-400, Republic of Korea

## Abstract

Recently, *γ*-synuclein (SNCG), which is also known as breast cancer-specific gene-1, has been demonstrated to be an adverse and aggressive marker in breast cancer. In our previous study, SNCG was significantly upregulated in irradiated human breast cancer cells. The aim of this study was to investigate whether radiation-induced, tumor-derived SNCG can influence dendritic cell (DC) function in immune systems. The phenotypical and functional changes of DCs in the presence or absence of SNCG were investigated by FACS analysis, ELISA, and real-time PCR. The ability of SNCG-treated DCs to influence T cells was also examined by coculturing with T cells. The treatment of DCs with SNCG protein inhibited the surface expression of the co-stimulatory molecules CD40 and CD86, and decreased the mRNA levels of pro-inflammatory cytokines. The SNCG-treated DCs inhibited T-cell proliferation slightly, but distinctively increased the population of regulatory T cells. In addition, the production of TGF-*β* from T cells was significantly increased when they were cocultured with SNCG-treated DCs. Taken together, these results demonstrate that tumor-derived SNCG contributes to immunosuppressive effects via the inhibition of DC differentiation and activation, thus making it a potential target for cancer treatment.

## Introduction

Radiotherapy (RT) is a well-established standard tumor treatment, and over half of all cancer patients will receive RT as part of their treatment plan.^1,2^ Exposure to ionizing radiation (IR) provokes several distinct cell death programs, such as apoptosis, necrosis, mitotic catastrophe, and autophagy, against tumor cells, as well as the surrounding immune cells.^3^ It is generally recognized that IR can cause suppressive and tolerogenic immune responses.^4–6^ However, an emerging body of evidence in recent years has suggested that the effects of IR on the immune system are complex. Ma *et al.* reported that IR induced ‘danger signals’ from dying tumor cells that may contribute to incite a potent antitumor immune response via immunogenic cell death (ICD) and reverting the immunosuppressive tumor microenvironment.^7–9^ However, the interplay between danger signaling patterns behind the trafficking of damage-associated molecular patterns (DAMPs) and their immune sensing systems appears to be very plastic and highly dependent on the dose and fractionation of radiation, the type of radiation-induced cell death, and the experimental conditions. Thus, whether the effect of intracellular proteins released by RT in cancer therapy could be beneficial or detrimental remains controversial.

We have recently demonstrated that single or fractionated doses of radiation induced several secretory proteins in human breast cancer cells.^10^ One of the interesting candidates from the previous study, *γ*-synuclein, was markedly increased by a high single dose of 10 Gy but not by fractionated irradiation. The synuclein family consists of three known members, *α*-, *β*-, and *γ*-synuclein (SNCA, SNCB, and SNCG, respectively). They are an unfolded group of proteins that is characterized by a 5–6 repeat (KTKEGV) consensus sequence in the first 87 residues of the N-terminal domain, whereas their acidic C-terminal domain is considerably different, likely to distinguish their function.^11^ SNCA and SNCB are located in neuronal cells, especially, the substantia nigra, subthalamic nucleus, amygdala, hippocampus, and thalamus, and have relevance to neurodegenerative disorders, such as Parkinson’s disease and Alzheimer’s disease.^12–14^ However, SNCG was cloned from infiltrating breast carcinoma cells and was not closely linked to neuronal degenerative disease.^15^ Several studies have revealed that SNCG is highly expressed in several cancer types, such as the advanced stages of breast, liver, ovarian carcinomas, colon, and prostate cancer.^16–20^ SNCG was shown to promote cell growth and motility that led to cancer metastasis and invasiveness.^21^ In addition, the overexpression of SNCG accelerated breast and ovarian cancer development.^17,22^ Therefore, whether the release of dying cancer cell-derived SNCG after RT could subsequently elicit anti-tumorigenic immunity or a pro-tumorigenic immune response remains unclear.

Dendritic cells (DCs) are distributed in epithelial tissues, including the lymphoid organs, skin, digestive, stromal and interstitial space of most parenchymatous organs. DCs play a role in immune homeostasis under physiological conditions and regulate immune activation during infection. DCs act as immune sentinels to initiate T-cell responses against microbial pathogens or tumors or to induce self-tolerance in the immature state.^23,24^

The generation, maturation, and function of DCs are all markedly inhibited in cancers. Defective function of these cells is considered one of the important factors responsible for tumor escape from immune surveillance.^25–27^ In contrast, the presence of dying tumor cells following RT promotes DC phagocytosis of the tumor cells, the processing of tumor-derived antigens, and the enhancement of antigen presentation and cross-presentation by DCs, resulting in a more robust adaptive immune response for anticancer activity.^28^ Therefore, DCs can exhibit various states and perform different functions depending on the environment they encounter. Based on the controversy regarding the effects of anticancer therapy on immune responses, the cross talk between radiation-induced changes in the tumor microenvironment and the maturation status of DCs after RT require clarification. In this study, we aim to investigate how the newly identified secretory SNCG derived from RT-treated dying tumor cells could affect DC maturation status. This study will provide clues to design a future rational and optimal regimen of RT for clinical application.

## Results

### SNCG was increasingly secreted by breast cancer cells exposed to IR

**Figure 1:**
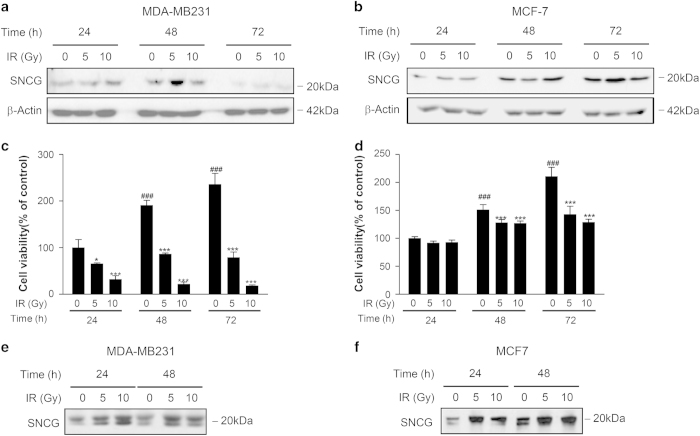
SNCG was increased by irradiated breast cancer cell lines. (**a** and **b**) Human breast cancer cell lines (MDA-MB231 and MCF-7) were exposed to 5 or 10 Gy doses of *γ*-irradiation, and the expression of SNCG was detected by western blotting at 24, 48 and 72 h after radiation. (**c** and **d**) The viability of MDA-MB231 and MCF-7 cells was determined by an MTT assay. (**e** and **f**) The secreted SNCG was detected in the conditioned media from MDA-MB231 and MCF-7 cells using immunoblotting. The data represents the mean ± S.D. of three independent experiments. ^###^ *P*<0.001 was compared with 24h 0 Gy group; while ***P<0.001 and *P<0.05 were compared with the 0 Gy group in their seperate time frames. Significance was determined by one-way ANOVA followed by Tukey's multiple comparison tests.

Many studies have shown that SNCG is overexpressed in advanced infiltrating breast carcinoma.^20^ We also found that SNCG was increased by radiating breast cancer cells using quantitative proteomic analysis.^10^ Here MDA-MB231 and MCF-7 human breast carcinoma cells were exposed to single doses of 5 or 10 Gy of radiation and harvested at the indicated times to investigate whether SNCG expression is increased by irradiation. The SNCG expression was increased in a dose-dependent manner in MDA-MB231 cells compared with the control group at 24 h, and the highest SNCG expression was observed at 48 h after irradiation with 5 Gy (Figure 1a). Similarly, the level of SNCG in MCF-7 cells was also increased by radiation and was sustained up to 72 h after irradiation (Figure 1b). To confirm total cancer cell death by radiation, an MTT assay was performed under the same experimental conditions as above (Figures 1c and d). Exposure to IR significantly decreased the viability of MDA-MB231 cells in a dose- and time-dependent manner, whereas irradiation of MCF-7 cells resulted in the inhibition of cell proliferation rather than induction of cell death. Next, we investigated whether SNCG, known to be a secretory molecule, is released from irradiated breast cancer cells. As shown in Figures 1e and f, SNCG was detected in conditioned medium, thus indicating that the induction and secretion of SNCG were mediated by irradiation of human breast cancer cells. Although these data did not exactly correspond with the expression profile of SNCG, this discrepancy may be because of the different amounts generated from endogenous or exogenous origin. These findings indicate that SNCG is markedly increased and secreted from irradiated breast cancer cells.

### SNCG inhibited the phenotypical maturation of bone marrow-derived DCs

**Figure 2:**
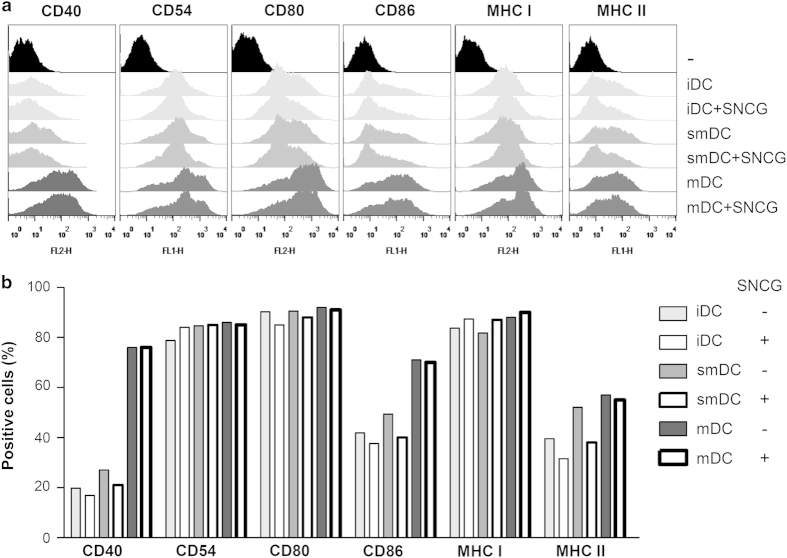
SNCG altered the phenotypic changes of DCs. BMDCs were generated by culturing with 10 ng/ml GM-CSF and 10 ng/ml IL-4 for 6 days. Then, the cells were treated with TNF-*α* (10 ng/ml) or LPS (1 *μ*g/ml) in the absence or presence of SNCG (1 *μ*g/ml) for 24 h. (**a**) The antigen-presenting (MHC-I and –II) and representative co-stimulatory molecules of DCs, including CD40, CD80, and CD86, and the CD54 adhesion molecule, were evaluated by flow cytometry. (**b**) The above histogram data were analyzed and depicted as a bar graph of positively stained cells.

The distinction between iDCs and mature DCs (mDCs) is based on phenotypical and functional changes. The enhancement of the antigen-presenting molecules MHC classes I and II along with the co-stimulatory molecules CD80 and CD86 is observed when DCs undergo maturation.^29^ iDCs finally differentiate into mDCs through semi-mDCs (smDCs) over time when continuously exposed to TNF-*α* or lipopolysaccharide (LPS). Based on these studies, we investigated the phenotype of three DC subsets (iDC, smDC, and mDC) in the presence or absence of SNCG. SNCG did not induce the apoptosis of bone marrow-derived DCs (BMDCs) during the GM-CSF/IL-4-mediated differentiation process (data not shown). As shown in Figure 2a, the expression of the co-stimulatory molecules CD40 and CD86 on mDCs were significantly increased compared with those on iDCs or smDCs. The antigen-presenting receptors MHC-I and -II, and an adhesion molecule, CD54, were also increased in mDCs. However, only minimal phenotypical changes between iDCs and smDCs were observed.

The expression of CD40, CD80, CD86, and MHC-II on smDCs or mDCs was downregulated when smDCs or mDCs were treated with SNCG (Figure 2b). These results indicate that SNCG impairs the antigen-presenting and T-cell-priming capacities of DCs by decreasing the expression of these surface molecules.

### SNCG prevented the functional maturation of BMDCs

**Figure 3:**
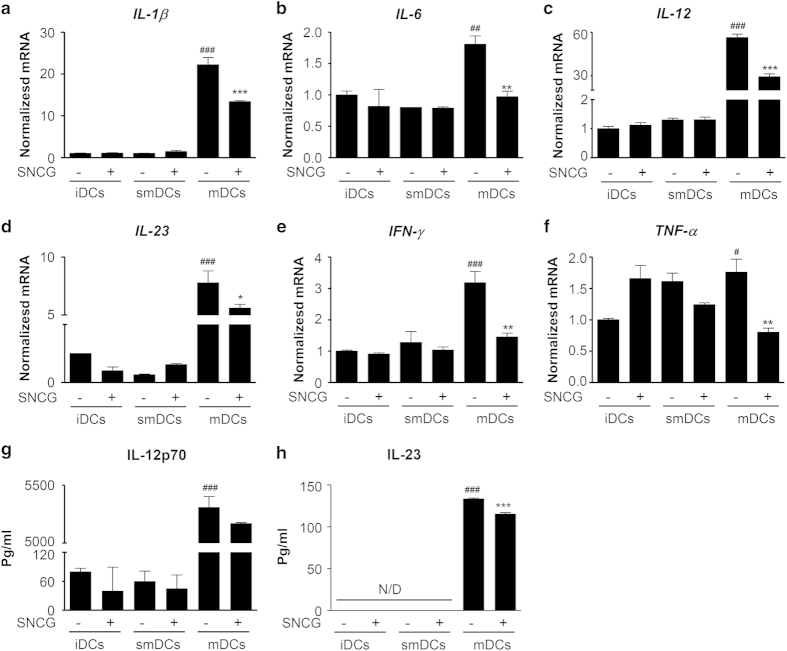
SNCG decreased immunostimulatory cytokines from mDCs. The iDCs, smDCs, or mDCs were treated with SNCG (1 *μ*g/ml) for 24 h. (**a**–**f**) The mRNA levels of IL-1*β*, IL-6, IL-12, IL-23, IFN-*γ,* and TNF-*α* were normalized to the 18S mRNA level. (**g** and **h**) The culture supernatants were evaluated for the production of IL-12p70 and IL-23 by ELISA. The data represent the mean±S.D. of three independent experiments. ^###^*P*<0.001, ^##^*P*<0.01 and ^#^*P*<0.05 were compared with the imDC group; ***P<0.001, **P<0.01 and *P<0.05 compared with the mDC group. Significance was determined by a one-way ANOVA followed by Tukey’s multiple comparison tests.

Next, we investigated the mRNA expression profile of several cytokines secreted from DC subsets in the presence or absence of SNCG to confirm the functional changes of the DC subsets. As shown in Figures 3a and f, the inflammatory cytokines were significantly increased in mDCs compared with those secreted by iDCs or smDCs, whereas we did not observe differences in cytokine secretion between iDCs and smDCs. The expression levels of all of the above-mentioned cytokines were significantly decreased in SNCG-treated mDCs compared with mDCs without SNCG. Furthermore, SNCG-treated mDCs produced a slightly reduced amount of IL-12p70 and IL-23 compared with mDCs without SNCG, as confirmed by an ELISA assay (Figures 3g and h). These results demonstrate that SNCG attenuated the production of pro-inflammatory cytokines of mDCs.

### SNCG-treated DCs generate anti-inflammatory and immunosuppressive T cells

**Figure 4:**
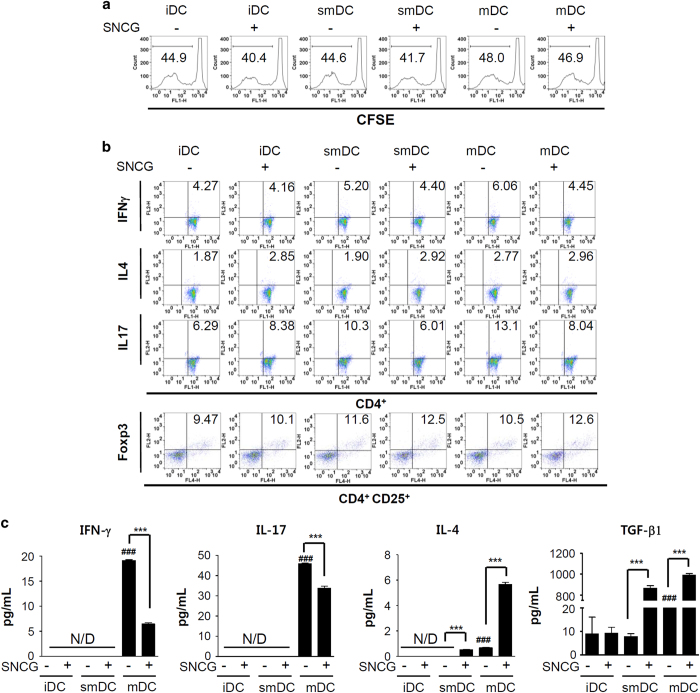
SNCG exhibits an immunosuppressive effect on DCs cocultured with CD4^+^ T cells. Spleens were isolated from 6- to 8-week-old BALB/c mice, and CD4^+^ T cells were isolated using a pluriBead KIT (pluriSelect, Deutscher Pl). (**a**) TNF-*α* (10 ng/ml) or LPS (1 *μ*g/ml) stimulated DCs were treated with SNCG (1 *μ*g/ml) and cocultured with CFSE-labeled CD4^+^ T cells (1 : 10) in 96-well round-bottom plates for 5 days. The proliferation of the T cells was determined by flow cytometry. Representative data are shown as histograms. (**b**) The differentiated DCs in the presence or absence of SNCG were cultured with T cells (1 : 10) in six-well plates for 5 days, and then the populations of IFN-*γ*-, IL-4-, and IL-17-secreting CD4^+^ T cells, as well as CD4^+^CD25^+^Foxp3^+^ Treg cells, were determined by flow cytometry. The number in each panel indicates the percentage of double-positive cells. One of three similar results is shown. (**c**) The supernatants from coculturing DCs and T cells were evaluated for IFN-*γ*, IL-4, IL-17, and TGF-*β* production by ELISA. The data represents the mean ± S.D. of three independent experiments. ^###^*P*<0.001 compared with the imDC group; ***P<0.001 compared with SNCG negative DC group. Significance was determined by a one-way ANOVA followed by Tukey's multiple comparison tests.

To examine the effect of SNCG-treated DCs on T cells, different subsets of DCs in the presence of SNCG were mixed with CFSE-labeled T cells for 5 days. The proliferation of T cells incubated with SNCG-treated DCs was decreased compared with T cells incubated with DC subsets without SNCG (Figure 4a). Next, to investigate the differentiation of T-cell subsets, the population of T cells producing IFN-*γ*, IL-4, or IL-17 was analyzed by flow cytometry following the T cell and DC coculture. smDCs mediate an increase in the population of IL-17-releasing CD4^+^ T cells, whereas mDCs activate CD4^+^ T cells generating IFN-*γ*, IL-4, or IL-17 (Th1, Th2, and Th17). Whereas SNCG-treated iDCs increased the population of IL-17- or IL-4-producing T cells, smDCs and mDCs treated with SNCG appeared to reduce the population of CD4^+^ T cells secreting IFN-*γ* or IL-17 cytokines. To determine the proportion of regulatory T cells (Tregs) in the coculture of T cells and SNCG-treated DCs, the total T cells were stained with anti-CD4, anti-CD25, and anti-Foxp3. The Treg cell population in all SNCG-treated DC cocultures was increased compared with the cells cocultured with SNCG-untreated DC subsets (Figure 4b). These data clearly indicate that SNCG-treated DCs increase the immunosuppressive Treg cell population when cocultured with T cells.

As shown in Figure 4c, the production of IFN-*γ* and IL-17 was significantly downregulated when T cells were cocultured with SNCG-treated mDCs; in contrast, IL-4 was markedly upregulated. Furthermore, a representative immunosuppressive cytokine, TGF-*β*, was also significantly increased in the supernatant of an SNCG-treated mDC (or smDC) and T-cell coculture. Taken together, SNCG-treated DCs induced Th2 immunity, consequently decreasing inflammatory cytokines and inhibiting antitumor immunity.

### Irradiated tumor cells inhibit DC activation

**Figure 5:**
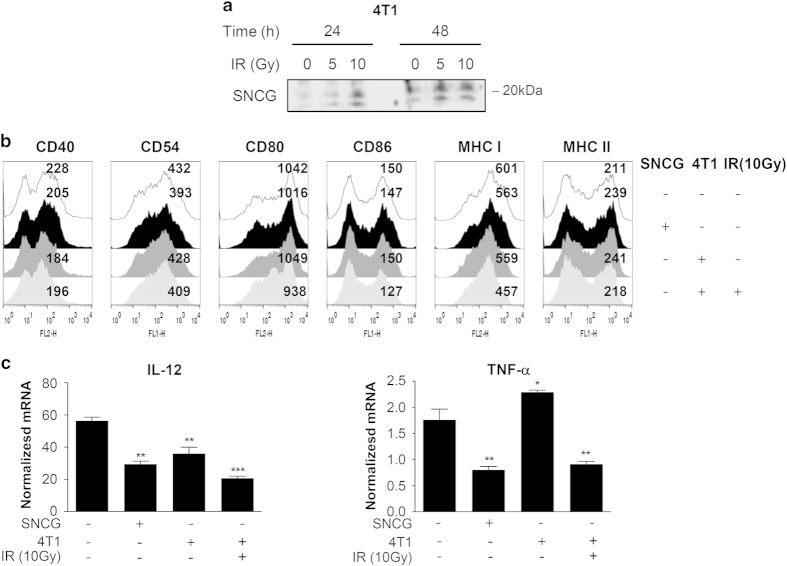
Irradiated tumor cells decreased DC maturation and activation. (**a**) The secreted SNCG was detected in the conditioned media from murine mammary 4T1 tumor cells using immunoblotting. (**b**) The mDCs (1×10^6^ cells) were stimulated with SNCG (1 *μ*g/ml) or irradiated 4T1 murine breast carcinoma cells (10 Gy, 2×10^5^ cells) for 48 h in a Transwell system. The surface maturation markers of the DCs were determined by FACS analysis. (**c**) The expression of IL-12 and TNF-*α* mRNAs isolated from DCs cocultured with SNCG or irradiated 4T1 tumor cells was investigated by real-time reverse transcription PCR and was normalized to 18S mRNA. The data represents the mean ± S.D. of three independent experiments. ***P<0.001, **P<0.01 and *P<0.05 compared with the mDC group. Significance was determined by a one-way ANOVA followed by Tukey's multiple comparison tests.

Because 4T1 is a murine mammary tumor cell line, we first investigated whether irradiated 4T1 cells also release SNCG. As shown in Figure 5a, the SNCG concentration was increased in the conditioned medium of irradiated 4T1 cells, similar to the results of Figures 1e and f. Next, to confirm whether soluble factors released by irradiated breast cancer cells were involved in the immunosuppressive activity of DCs, iDCs were separated from irradiated 4T1 cells by Transwell system with a membrane pore size of 0.4 *μ*M, which enables only soluble factors to diffuse. The 4T1 tumor cells did not further increase the maturation marker expression of mDCs, whereas the antigen-presenting molecules MHC-I and MHC-II and the co-stimulatory molecules CD80 and CD86 were markedly reduced on DCs stimulated by irradiated 4T1 cells (Figure 5b). In addition, the IL-12 and TNF-*α* mRNA expression from mDCs stimulated with irradiated 4T1 cells was significantly decreased compared with non-treated mDCs or mDCs stimulated with non-irradiated 4T1 cells, similar to those of SNCG-treated DCs (Figure 5c). Taken together, these results indicate that irradiated breast cancer cells can release several factors containing SNCG, thus impairing DC maturation via the downregulation of surface maturation markers and immunostimulatory cytokines.

## Discussion

DCs have a vital role as professional APCs that are able to activate naive T cells and initiate T-cell responses, acting as messengers between the innate and adaptive immune systems.^30^ Therefore, many researchers have been highly interested in regulating DC activity and exploring DCs as therapeutic targets for treating various inflammatory and immunosuppressive disorders.^31^ RT has traditionally been recognized as cytotoxic and immunosuppressive. Recently, substantial evidence has prompted the re-characterization of radiation as immunomodulatory rather than immunosuppressive. Thus, there have been many efforts to investigate the integration of RT in immune-based therapies. RT enhances the expression of tumor-associated antigens and secret immuno-activating danger signals, such as high-mobility group protein B1, heat-shock proteins, calreticulin, ATPs, and hepatoma-derived growth factor; thus, tumors can be easily detected by the immune system.^32^ Given our previous findings that SNCG was increased and released by irradiated breast cancer cells, we considered the possibility of SNCG as a DAMP, and thus investigated the effect of SNCG on the phenotypical and functional alteration of murine DCs.

Upon exposure to SNCG, TNF-*α*- or LPS-stimulated smDCs or mDCs reduced their expression of several surface molecules that contribute to co-stimulation and antigen presentation to T cells. In addition, mDCs in the presence of SNCG significantly reduced the production of the inflammatory cytokines IL-1*β*, IL-6, IL-12, IL-23, IFN-*γ*, and TNF-*α*. Although smDCs have been reported to induce Treg cells and reduce the levels of pro-inflammatory cytokine expression, smDCs might be inconsistent either in the upregulation of phenotypic maturation ligands or in the secretion of cytokines depending on the experimental conditions, and thus it is difficult to distinguish them from mDCs in this study. Coculture with SNCG-treated DCs downregulated T-cell proliferation and altered the T-cell cytokine production profile, reducing pro-inflammatory cytokine IFN-*γ* and IL-17 secretion and inducing the anti-inflammatory cytokines IL-4 and TGF-*β*. IR has been shown to release TGF-*β in vitro* and *in vivo*. Whereas TGF-*β* leads to growth inhibition and apoptosis, and serves as a tumor suppressor gene in normal tissues, TGF-*β* expression is often increased and can be involved in some oncogene-like function in many tumors. We found that SNCG can significantly enhance TGF-*β* production by smDCs and mDCs. Owing to limitations in our ability to evaluate the *in vivo* quantitative and qualitative DC activation in the tumor microenvironment, we further investigated whether the soluble secretory factors from irradiated tumor cells may actually affect DC maturation, similar to SNCG. Similarly, irradiated tumor cells inhibited the activation of LPS-stimulated DCs through a decrease in surface maturation ligands and inflammatory IL-12 and TNF-*α* cytokine production. In summary, SNCG derived from RT-treated dying tumor cells may moderate the stimulation of DCs, similar to smDCs, with low expression of phenotypic maturation ligands and the induction of immunosuppressive cytokines, thereby rendering the DCs incapable of efficiently interacting with T cells or eliciting fully immunogenic responses.

ICD has been demonstrated to be a cascade in which dying tumor cells release immunogenic factors that are received and processed by DCs, which in turn present antigen to activated cytotoxic T lymphocytes. However, whether RT specifically and efficiently elicits ICD remains a critical research question. Whereas RT may be efficient at releasing tumor antigens, the immunosuppressive tumor microenvironment may hamper the development of therapeutically effective antitumor immune responses.^33^ Furthermore, the DAMP spectrum can change even for the same cancer cell line depending on the type of treatment; the optimal dosing, timing, and sequencing of RT or other stimuli must be further investigated. Moreover, the ability of DCs to prime T cells could be influenced by the concentration of antigenic peptide–MHC complex per DC; a higher concentration allows for more rapid T-cell activation.^34,35^ Consistent with these findings, the DC subsets did not change under treatment with 5 *μ*g/ml. Because we did not investigate the effect of a broad range of concentration SNCG on DCs, it remains possible that a higher concentration of SNCG may inversely cause an activation of DCs. However, the single 10 Gy dose of radiation was sufficient to release SNCG from dying cancer cells and evoked an immunosuppressive response against tumors.

Despite the accumulation of emerging evidence, it still remains challenging to understand how, when, and to what extent this dynamic spectrum of DC activation drives tumor-specific antitumor immunity, particularly, in the context of anticancer therapy. In this respect, the pre-existing or therapy-generated tumor microenvironments, as well as the cross talk between dying cancer cells and DCs, mediated by soluble and vesicular factors, are crucial determinants of the DC maturation status and anticancer immune response. Furthermore, the complex interplay between the radiation dose and the effect on target tissue-associated factors and host immunity has yet to be consistently defined. Despite these limitations, our findings indicate that SNCG, which can be released from dying irradiated breast cancer cells, might be at least partially involved in the persistence of tumor resistance against RT, and modulation of SNCG may be a promising approach for anticancer therapy.

With emerging interest in studying the mechanisms of IR-induced ICD, it is necessary to find novel immunomodulators and analyze certain existing therapies for their potential to cause DC maturation irrespective of whether they induced ICD. This study also cautiously suggests the predictable response of DCs against radiation-induced dying cancer cells.

## Materials and Methods

### Mice

Female BALB/c mice, 6–8 weeks of age, were purchased from Samtako Bio Korea (Osan, Korea). All animal experiments were conducted according to the Korean National Guidelines of Laboratory Animal Experiment based on the protocol approved by the Institutional Animal Care and Use Committee of the Korea Institute of Radiological and Medical Sciences (KIRAMS 13-056).

### Cell cultures and generation of BMDCs

BMDCs were generated as previously described^36–40^ with some modifications. Briefly, bone marrow cells were harvested from the femur and tibia of the hind legs. Next, the cells were depleted of red blood cells (RBCs) with RBC lysis buffer (Lonza, Walkersville, MD, USA). The cells were cultured for 6 days in RPMI 1640 (Lonza) containing 10% fetal bovine serum (Gibco, Grand Island, NY, USA), 10 000 units/ml penicillin (Gibco), 10 ng/ml rmGM-CSF (JW CreaGene, Daegu, Korea) and 10 ng/ml rmIL-4 (JW CreaGene, Daegu, Korea). The culture medium was replaced every 2 days. After 7 days, DCs were incubated for 24 h with 10 ng/ml rmTNF-*α* (PeproTech, Rocky Hill, NJ, USA) or 1 *μ*g/ml LPS (Sigma-Aldrich, St. Louis, MO, USA) to generate smDCs or fully mDCs, respectively. At the same time, 1 *μ*g/ml rhSNCG (Acris, San Diego, CA, USA) was co-administered to differentiate the DCs to investigate the effect of SNCG. The purity of the BMDC population was assessed by flow cytometry after CD11c labeling (>90% for CD11c^+^ cells).

### Western blot analysis

Cells (1×106 cells per ml) cultured in 100 mm plates (SPL Lifescience, Pocheon, Korea) were lysed with RIPA buffer (50 mM Tris–HCl (pH 7.4), 150 mM NaCl, 1% NP-40, and 1% sodium deoxycholate, and containing 1 mM EDTA) supplemented with protease inhibitors (1 mM PMSF, 1 *μ*g/ml aprotinin, 1 mM Na_3_VO_4_ and 1 mM NaF). Proteins from whole-cell lysates were separated on 8–15% SDS-polyacrylamide gels and transferred to nitrocellulose membranes (Bio-Rad, Berkeley, CA, USA). The membrane was blocked with 5% skimmed milk in Tris-buffered saline containing 0.1% Tween-20 (TBS-T) for 1 h and probed with anti-SNCG (Abcam, Cambridge Science Park, Cambridge, UK) or anti-*β*-actin antibodies (AbFrontier, Seoul, Korea) overnight at 4 °C. After multiple washes, the membranes were incubated with peroxidase-conjugated secondary antibodies (Thermo Scientific, Waltham, MA, USA), and immunoreactive bands were detected using enhanced chemiluminescence reagents according to the manufacturer’s recommendations (GE Healthcare, Little Chalfont, UK). The experiments were repeated at least three times. The immunoreactive bands were semi-quantified using a densitometer (Bio-Rad) and normalized to the band intensity of *β*-actin as a loading control.

### Flow cytometry

Immunostaining was performed as described previously.^41^ Briefly, after 10 min of incubation at 4 °C with Fc receptor blocker (BD Biosciences, San Joses, CA, USA), the cells were stained with the corresponding antibodies. The antibodies used for DC phenotyping were CD11c-PE, CD14-FITC, CD40-PE, CD54-FITC, CD80-PE, CD86-FITC, H-2K[d]-PE, and I-A[d]-FITC; all these antibodies were purchased from BD Biosciences. For intracellular cytokine staining, the cells were incubated with brefeldin A (BD Biosciences) for 1 h. The cells were stained with surface markers, such as CD4-FITC and CD25-APC (BD Biosciences). Then, the cells were permeabilized using Cytofix/Cytoperm reagents (BD Biosciences) and stained for intracellular markers as follows: IFN-*γ*-PE, IL-4-PE (BD Biosciences), IL-17A-PE, and Foxp3-PE (eBioscience, San Diego, CA, USA). Stained cells were acquired using a BD FACSCanto II flow cytometer (BD Biosciences) and analyzed using the FlowJo software (v.10, Ashland, OR, USA).

### Measurement of cytokines

Cell-free supernatants derived from DCs alone as well as coculture with DC and T cells were harvested, and the cytokine levels were determined by ELISA. The amounts of IL-12p70, IFN-*γ*, IL-4 (BD Biosciences), IL-17A, TGF-*β* (R&D Systems, Minneapolis, MN, USA), or IL-23 (Invitrogen, Waltham, MA, USA) ELISA kits were evaluated according to the manufacturer’s instructions.

### Real-time quantitative reverse transcription PCR

Total RNA was isolated from DCs using TRIzol (Invitrogen Life Technologies, Waltham, MA, USA), and RNA was reverse transcribed using M-MLV Reverse Transcriptase (Enzynomics, Daejeon, Korea), Oligo dT (Enzynomics) and RNasin Ribonuclease Inhibitor (Promega, Fitchburg, WI, USA). The mRNA levels were assessed by real-time quantitative reverse transcription PCR using Maxima SYBR Green quantitative PCR Master Mix (Thermo Scientific) with a CFX96 Touch Real-Time PCR Detection System (Bio-Rad). Relative quantification of gene expression was performed using the ΔΔ*C*_t_ method in CFX Manager software (Bio-Rad) using 18S ribosomal RNA as an internal reference. The primer pairs for the analyzed genes were supplied by Bioneer (Daejeon, Korea), and the sequences were as follows: IL-6 (N-4013); IL-1*β* (N-4009); TNF-*α* (N-4015); 18S forward 5′-CGAAAGCATTTGCCAAGAAT-3′, reverse 5′-AGTCGGCATCGTTTATGG TC-3′; IL-12 forward 5′-CTCCTGTGGGAGAAGCAGAC-3′, reverse 5′-CAGATAGCC CATCACCCTG-3′; IL-23 forward 5′-AATAATGTGCCCCGTATCCA-3′, reverse 5′-AGGCTCCCCTTTGAAGATGT-3′; IFN-*γ* forward 5′-GAGGTCAACAACCCACAGGT-3′, reverse 5′-GGGACAATCTCTTCCCCACC-3′.

### Coculture of DCs and T cells

DCs (2×10^5^ cells per ml) were cocultured with CD4^+^ T cells (1×10^6^ cells per ml), which were isolated from splenocytes obtained from naive BALB/c mice using a CD4 isolation kit (pluriSelect, Deutscher Pl, Leipzig, Germany). The cells were cocultured in RPMI 1640 supplemented with 10% FBS for 72 h at 37 °C with 5% CO_2_. To evaluate T-cell proliferation, isolated CD4^+^ T cells were stained using a CellTrace CFSE Cell Proliferation Kit (Life Technologies, Waltham, MA, USA) and analyzed by flow cytometry. The DCs (2×10^5^ cells per ml) were transferred to a 96-well plate and then cocultured with CFSE-labeled T cells (2×10^6^ cells per ml) for 5 days at 37 °C with 5% CO_2_.

### DC sensitization with irradiated tumor cells

To determine the effect of secretory molecules from irradiated tumor cells on DCs, 4T1 murine mammary carcinoma cells (2×10^5^) were cocultured with DCs using a Transwell system. The LPS-stimulated mDCs (1×10^6^) were seeded in 24-well plates. The 4T1 murine mammary carcinoma cells (2×10^5^) were exposed with or without a 10 Gy dose of radiation and loaded in 0.4-*μ*m pore size Transwell inserts on a 24-well cell culture plate (Corning Glass, Seoul, Korea). Control DCs were cultured using the same conditions but in the absence of 4T1 cells.

### Statistical analysis

Statistical analyses were performed using the GraphPad software, version 5 (GraphPad, La Jolla, CA, USA). The data are presented as mean±S.D. Significant differences between the groups were determined by analysis of variance and Tukey’s *post hoc* test. *P-*values <0.05 were considered statistically significant.
